# Probing the structure of D_2_O ice layers on ALD-grown ZrO_2_, Al_2_O_3_ and TiO_2_ thin films by sum frequency generation (SFG) spectroscopy

**DOI:** 10.1039/d5fd00152h

**Published:** 2025-12-16

**Authors:** Xia Li, Susanne Gross, Thomas Haunold, Moon-Hyung Jang, Marketa Zukalova, Martin Jindra, Joanna E. Olszówka, Yu Lei, Štefan Vajda, Günther Rupprechter

**Affiliations:** a Institute of Materials Chemistry, TU Wien Vienna 1060 Austria xia.li@tuwien.ac.at guenther.rupprechter@tuwien.ac.at; b Department of Chemical and Materials Engineering, University of Alabama in Huntsville Huntsville AL 35899 USA; c Department of Electrochemical Materials, J. Heyrovsky Institute of Physical Chemistry Dolejškova 2155/3 182 00 Prague 8 Czech Republic; d Department of Nanocatalysis, J. Heyrovsky Institute of Physical Chemistry Prague Czech Republic

## Abstract

Sum frequency generation (SFG) spectroscopy was applied to investigate D_2_O adsorption on atomic layer deposition (ALD)-grown Al_2_O_3_, ZrO_2_, and TiO_2_ films at 94 ± 1 K. Film composition and thickness were characterized by ellipsometry and X-ray photoelectron spectroscopy (XPS). Additional SFG measurements were conducted on the SiO_2_/Si wafer and on a CoO film prepared by oxidizing Co foil. At D_2_O exposure below 3000 L, the spectra were dominated by interfacial features originating from the ice–oxide interface. These spectra exhibited a weak, broad O–D stretching band (OD_3_) centered at 2650 cm^−1^, tentatively attributed to (dissociated) water molecules hydrogen-bonded to the oxide surface; this assignment was supported by the absence of the OD_3_ feature on the SiO_2_/Si substrate. A sharp peak at 2730 cm^−1^ was also observed and assigned to the “free” O–D stretch (non-hydrogen-bonded with any neighboring molecule) of surface D_2_O molecules pointing into the vapor phase. Upon increasing D_2_O exposure, both the OD_3_ and “free” OD bands decreased in intensity and were replaced by weakly hydrogen-bonded OD_2_ and strongly hydrogen-bonded OD_1_ modes associated with the ice–vapor interface. As the exposure increased further, the OD_2_ and OD_1_ bands shifted to lower wavenumbers (2310 to 2284 cm^−1^) and became stronger, with the OD_1_ mode exhibiting a larger red shift and more pronounced intensity enhancement. No significant differences in water structure were observed on the Al_2_O_3_, ZrO_2_, and CoO films at the ice–vapor interfaces, apart from an approximately fivefold reduction in intensity on CoO, which is attributed to signal scattering from the rough CoO film/Co foil surface. However, when D_2_O exposure reached ≥30 000 L, the OD_1_ band on the TiO_2_ surfaces decreased substantially in intensity and shifted to much lower wavenumbers (2065 cm^−1^ at 30 000 L; 2030 cm^−1^ at 102 000 L) than on Al_2_O_3_ (2283 cm^−1^ at 90 000 L), ZrO_2_ (2293 cm^−1^ at 30 000 L), and CoO (2284 cm^−1^ at 900 000 L), indicating specific hydrogen-bonding interactions on the TiO_2_ surface.

## Abstract 2

Surface-specific SFG was used to reveal interactions between D_2_O ice and various ALD-grown metal oxide films.

## Graphical abstract

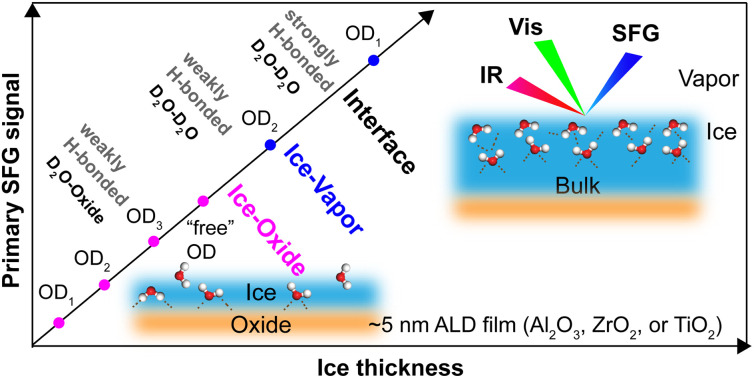

## Introduction

Water plays a pivotal role in numerous chemical, physical and biological processes, both in nature and industry. With the growing global concern over energy generation and environmental pollution associated with the extensive use of non-renewable fossil fuels, the development of clean and efficient hydrogen (H_2_) production technologies has become increasingly important. Among various approaches, water serves as a direct key source for H_2_ such as water–gas shift (WGS) (CO + H_2_O ↔ CO_2_ + H_2_, Δ*H* = −41.2 kJ mol^−1^)^1–5^ and methanol steam reforming (MSR) (CH_3_OH + H_2_O ↔ CO_2_ + 3H_2_, Δ*H* = +49.7 kJ mol^−1^),^6–8^ which are typically catalyzed by oxide-supported transition metals, or water serving as a promoter affecting activity and/or selectivity.^9–11^

Metal oxides play a crucial role in these processes, particularly in the activation and dissociation of H_2_O. Among commonly used supports such as CeO_2_, Al_2_O_3_, ZrO_2_, or TiO_2_, the catalytic activity varies significantly due to differences in strong metal–support interactions (SMSI).^3,4,12,13^ For instance, in the WGS reaction, Pt–Ni bimetallic catalysts supported on reducible or partially reducible oxides (CeO_2_, TiO_2_ and HSA-ZrO_2_) exhibit higher activity than those supported on non-reducible oxides (γ-Al_2_O_3_, SiO_2_ and LSA-ZrO_2_).^3^ Similarly, for Cu-based catalysts in WGS,^5^ CO conversion between 320 and 360 °C decreases in the order of Cu/CeO_2_ > Cu/MgO > Cu/ZrO_2_ > Cu/Al_2_O_3_.

In the case of MSR using Pd-based catalysts, Takezawa and co-workers^13^ compared various oxide supports and found that Pd/ZrO_2_ exhibits excellent MSR activity and selectivity, second only to Pd/ZnO. The overall activity order of the oxide supports was ZnO > ZrO_2_ > Nd_2_O_3_ > La_2_O_3_ > Al_2_O_3_ > Nd_2_O_5_ > SiO_2_. These observations clearly demonstrate the significant influence of the oxide support on catalytic performance.

Furthermore, water–oxide interactions are of great importance across various fields, including corrosion, catalysts, geochemistry, atmospheric chemistry, biology, and materials science, as illustrated in numerous review articles.^14–17^ Therefore, elucidating the structure and behavior of interfacial water on different oxide surfaces is of particular interest.

However, distinguishing between monolayer and multilayer water structures on oxide surfaces remains challenging, as several traditional surface characterization techniques have inherent limitations.^16^ Electron spectroscopies are hindered by the insulation of bulk water, which causes surface charging. Scanning tunneling microscopy can only be applied to films thinner than three molecular layers to avoid conductivity issues. Vibrational spectroscopic techniques, such as surface infrared spectroscopy, are well suited for probing adsorbate geometry and dynamics,^18^ however, infrared measurements generally average over both bulk and surface contributions.^16^

Sum frequency generation (SFG) spectroscopy,^19–22^ a second-order nonlinear optical technique, has proven particularly effective for probing molecular vibrations at surfaces/interfaces,^22–26^ as the adjacent bulk phases do not contribute to the signal. Using this method, numerous studies have investigated water structures at a variety of interfaces, including the water–air interface,^27^ water–metal interfaces,^28^ aqueous–mineral interfaces (*e.g.*, SiO_2_, Al_2_O_3_, CaF_2_, and TiO_2_),^29,30^ graphene–water,^31–33^ graphene oxide–water,^34^ and ultrathin Au film–water (2 nm or less) systems.^35^

Ice, a hydrogen-bonded solid form of water, consists of water molecules held together by a tetrahedral hydrogen bonding network. However, ice is structurally complex, exhibiting as many as 17 crystalline polymorphs and 2 amorphous solids.^36^ Its high vapor pressure (above 170 K, >10^−3^ Pa)^36^ and polymorphism make it a challenging subject in surface science. Shen and coworkers^37^ first reported conventional SFG spectra of the basal face of single-crystalline ice I_h_ at 170–270 K in 2001. Subsequently, ice at various surfaces and interfaces (*e.g.*, air, SiO_2_, Pt(111), MgO(001)) has been extensively studied using SFG.^36,38–41^

A single water layer can readily form at low temperatures (<150 K) under UHV conditions.^16^ Somorjai and coworkers^39^ demonstrated that ice films (<30 ML thickness) grown on Pt(111) at 120–137 K exhibit ferroelectric ordering—a term loosely describing a net polar orientation of water molecules within the ice films. Classified based on the number of hydrogen bonding donors (D) and acceptors (A), below 200 K, the topmost ice surface becomes increasingly crystalline, where double donor–double acceptor DDAA-type water molecules break one hydrogen bond and rearrange into the hexagonal H-bond network, forming single donor–double acceptor DAA-type water species.^40^ At the ice–sapphire interface, a sharp peak at 3100 cm^−1^ has been attributed to the OH stretching of highly ordered water molecules in ice I_h_ crystals.^31^ In contrast, the ice structure adjacent to graphite shows little to no temperature dependence between 261 and 273 K. Molecular dynamics (MD) simulations have further suggested that monolayer ice confined inside graphene nanocapillaries forms a puckered zigzag structure.^42^

In this study, we investigated D_2_O ice (amorphous solid water) adsorption at 93–95 K, a relatively low temperature, under various exposures rather than temperature variations, on three atomic layer deposition (ALD) oxide films—Al_2_O_3_, ZrO_2_, and TiO_2_—using a Si wafer as the substrate (as reported in ref. 43 and 44). Water adsorption on pure Si and on a CoO film supported on an unpolished Co foil are used as references. Water exposure is controlled by adjusting both the D_2_O vapor pressure and exposure time.

## Results and discussion

**Fig. 1:**
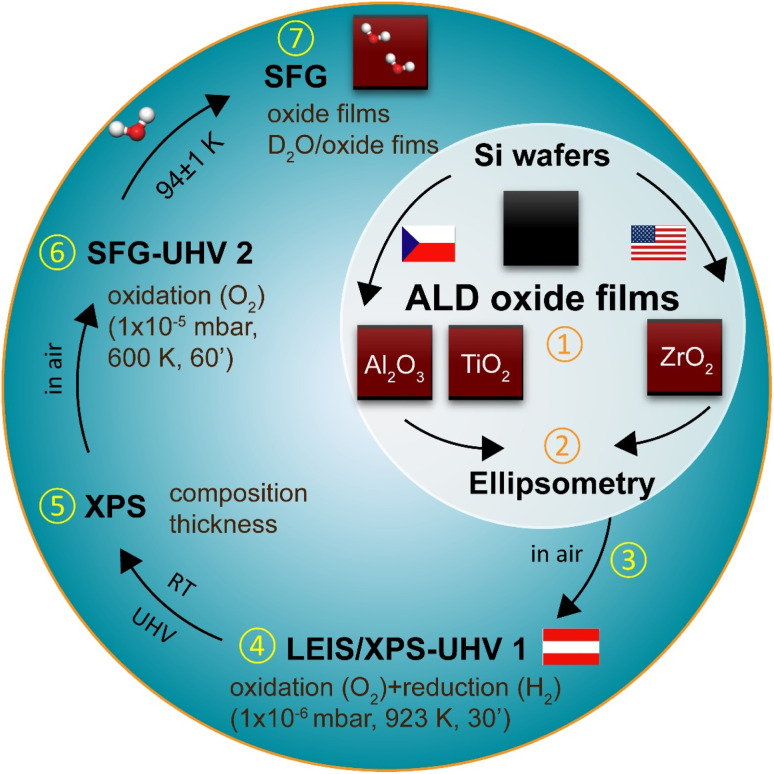
Schematic diagram of the ALD-sample preparation and characterization process.

Prior to the discussion of the results, the procedure of ALD sample preparation and characterization is briefly illustrated in Fig. 1. All atomic layer deposition (ALD) oxide films (Al_2_O_3_, TiO_2_, ZrO_2_) were grown on Si(100) wafers (size: 7 × 7 mm^2^) and the thicknesses were measured by ellipsometry. After transferring into the XPS/LEIS-UHV 1 system (Austria), the samples were thoroughly cleaned by oxidation (1 × 10^−6^ mbar O_2_, 923 K, 30 min) and reduction (1 × 10^−6^ mbar H_2_, 923 K, 30 min) to remove potential contaminations introduced by air exposure. All XPS measurements were performed at room temperature (RT) under UHV conditions. Subsequently, the ALD samples were transferred in air to the UHV-compatible SFG cell (SFG-UHV 2). Again, accounting for air exposure, the samples were oxidized (1 × 10^−5^ mbar, 600 K, 60 min) before SFG measurements to remove any remaining contaminants. To ensure clean surfaces, SFG spectra of the pure oxide films were recorded first. Finally, SFG spectra were obtained at 93–95 K after dosing water at the same temperature.

### Characterization of ALD oxide films by XPS and SFG

#### XPS

The oxide film thicknesses were measured by ellipsometry, yielding about 5.1 nm for Al_2_O_3_, 5.0 nm for TiO_2_, and 6.9 nm for ZrO_2_. However, in this thickness range, ellipsometry is neither as accurate as XPS, nor does it provide compositional information.

When exposed to air at room temperature, Si wafers naturally form a passivating SiO_2_ layer (*i.e.*, native (n-)SiO_2_; depending on exposure time and humidity), typically ranging from a few angstroms (Å) to several nanometers (nm).^45–47^ Accordingly, the ALD films were deposited on top of these n-SiO_2_ layers (in our case, ∼2.0 nm by ellipsometry).

The Si wafer substrate is best represented by the XPS Si 2p region, whereas the individual ALD films were characterized by the Al 2p, Zr 3d, and Ti 2p regions (Fig. S1). The binding energy positions and line shapes of Al 2p, Zr 3d and Ti 2p region spectra correspond well to literature values^48–50^ for Al_2_O_3_, ZrO_2_, and TiO_2_, respectively. Detailed fitting procedures are provided in the SI.

The film thicknesses of the ALD oxides as well as of the SiO_*x*_/n-SiO_2_ interlayers were calculated from the mentioned region spectra applying a multilayer electron attenuation model based on the Strohmeier approach.^51^ The corresponding results are summarized in Table 1.

**Table 1:** Binding energies and film thicknesses of all oxide films grown on Si wafer

Samples	Al_2_O_3_/SiO_*x*_/n-SiO_2_		ZrO_2_/n-SiO_2_		TiO_2_/n-SiO_2_	
	Al 2p	Si 2p	Zr 3d	Si 2p	Ti 2p	Si 2p
BE (eV)	74.1	99.3/101.8	181.9	102.2	458.9	102.2
Oxide film	Al_2_O_3_	SiO_*x*_/SiO_2_	ZrO_2_	SiO_2_	TiO_2_	SiO_2_
Thickness (nm)	4.9	≈1.1	4.3	1.1	4.3	1.1
Sum (nm)	6.0		5.4		5.4	

Ti and Zr have comparably high oxygen affinities (due to similar enthalpies of oxide formation) but react with SiO_2_ only at elevated temperatures (>900 K).^52,53^ As a result, the original n-SiO_2_ layer (1.1 nm) was largely preserved during ALD growth of TiO_2_ and ZrO_2_, producing a consistent Si 2p peak at 102.2 eV and an n-SiO_2_ thickness of 1.1 nm in both cases. In contrast, Al has an even higher oxygen affinity, enabling partial reduction of the n-SiO_2_ and formation of Si suboxides (“SiO_*x*_”, 99.3 eV). This caused a downward binding energy shift of the Si 2p region by 0.4 eV. Since nothing further can be said about the crystallography of SiO_*x*_, it is assumed as first approximation that the reduction of n-SiO_2_ has not significantly changed the thickness of the passivation layer (≈1.1 nm). Below, the n-SiO_2_ surface is simply referred to as “Si wafer”.

#### SFG: 2150–3000 cm^−1^

After the XPS measurements, the samples were transferred to the SFG cell for further characterization. The SFG spectra can be measured using the ssp or ppp polarization combinations.^19–22^ Here, s and p denote polarizations of the optical field perpendicular to and within the plane of incidence, respectively. They are listed in the order of relative beam energies (for example, s-SFG, s-visible and p-IR). Prior to SFG, all oxide films were pretreated to remove carbonaceous and/or hydrocarbon (organic) contaminants. As shown in Fig. S2, the C–H stretching peaks at 2800–3000 cm^−1^ decreased significantly after annealing in O_2_ (1 × 10^−5^ mbar O_2_, 600 K, 60 min) compared with annealing under UHV conditions. Therefore, for all subsequent SFG measurements, the oxide films were routinely pretreated by oxidation.

**Fig. 2:**
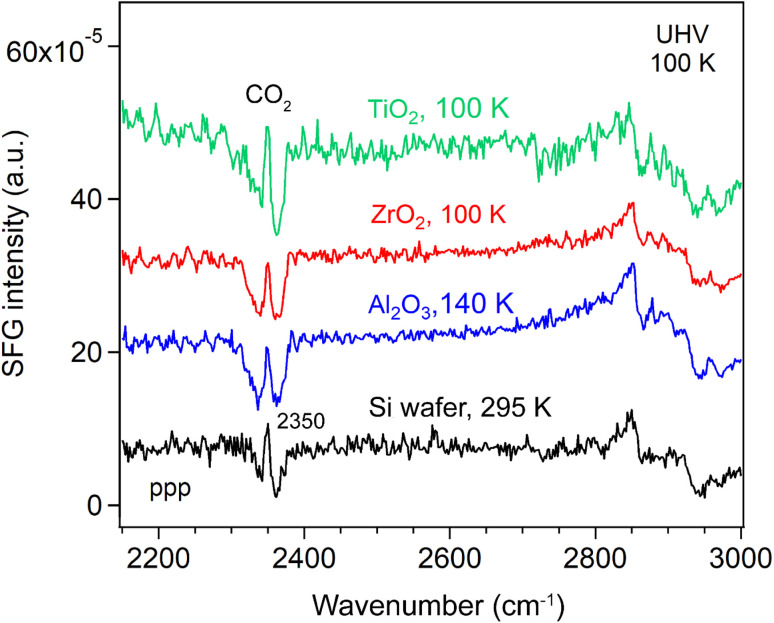
ppp-SFG spectra of Si wafer and ∼5 nm ALD films of Al_2_O_3_, ZrO_2_, and TiO_2_ under UHV, with offset for clarity. Temperatures are indicated.

We then measured the SFG spectra of the oxide films in the range of 2100–2800 cm^−1^ (Fig. 2), which includes the O–D stretching region (used as a reference for the D_2_O spectra) and C–H stretching region^54^ (used to evaluate residual organic contaminants) at 100/140 K. An SFG spectrum of the Si wafer at 295 K was also measured for reference. The non-resonant SFG responses from the Si wafer with an intrinsic ∼1.1 nm SiO_2_ passivation layer (black), as well as from the Si-supported Al_2_O_3_ (blue), ZrO_2_, (red) and TiO_2_ (green) films, were essentially identical. This indicates that all the metal oxide films (4.3–4.9 nm) produced no detectable non-resonant SFG signal, which is further supported by the distinctly different SFG response observed for a 60 nm TiO_2_ film.^55^

#### SFG: 3000–3800 cm^−1^

**Fig. 3:**
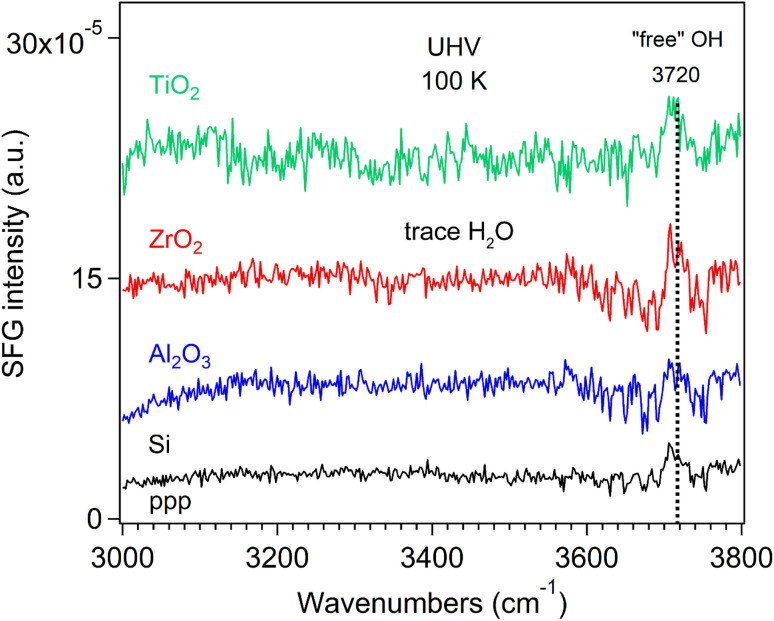
ppp-SFG spectra of oxide films at 100 K under UHV in the range of 3000–3800 cm^−1^.

We also measured the SFG spectra of neat Si wafer and Si-supported oxide films under UHV in the O–H stretching region (3000–3800 cm^−1^) at 100 K. Interestingly, a peak at 3720 cm^−1^ was observed (Fig. 3), which is attributed to the dangling O–H bonds pointing out of the ice at the surface, *i.e.*, the “free” O–H stretching mode, arising from trace water molecules present in the UHV chamber. The signal originates from the total population of water molecules in the DAA, DA, and AA configurations that exhibit “free” O–H groups.^40^ This “free” O–H peak disappeared upon heating to room temperature (Fig. S3).

Nagata and coworkers^40^ reported that the topmost monolayer of water on the basal face of ice (from 150 K to 245 K) exhibits a minimum number of “free” O–H groups and a maximum in hydrogen bonding around 200 K. Above 200 K, thermal fluctuations break hydrogen (H) bonds and generate more free O–H groups; below 200 K, the formation of bulk-like crystalline interfacial structures also results in H-bond breaking, thereby increasing the population of free O–H groups. At 300 K, H_2_O (D_2_O) was reported to be partially dissociated on the Si(111) surface to form the SiOH (SiOD) species.^56^ As the monolayer water desorption occurs at around 171 K and gradually moves to low temperature (150 K) with increasing the water thicknesss.^39^ Shen and coworkers^37^ found that the “free” OH band in both ssp and ppp polarization combinations becomes narrower with decreasing temperature (170–270 K), which they attributed to a narrowing of the orientational distribution of the O–H bond. Below 200 K, the “free” OH groups align almost perfectly upright with an assumption of a truncated flat distribution, indicating the absence of a quasi-liquid layer (QLL) or any surface layer with significant fluidity. Above 200 K, the onset and evolution of QLL introduce surface disorder.^37^

#### SFG spectra of D_2_O ice on a Si substrate and Si-supported oxide films in the O–D stretching region (2150–2800 cm^−1^)

##### Low D_2_O exposure

We initially measured SFG spectra of D_2_O adsorption at room temperature, but no signal was detected due to desorption.^39^ Consequently, we focused on spectra acquired at low temperature 94 ± 1 K, the minimum achievable with liquid N_2_ cooling.

**Fig. 4:**
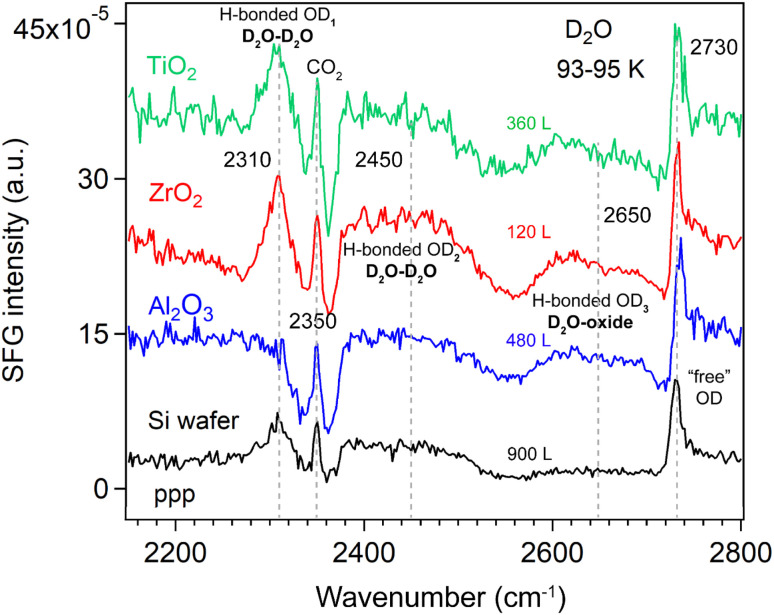
ppp-SFG spectra of D_2_O adsorption on Si wafer, Si-supported ∼5 nm ALD films of Al_2_O_3_, ZrO_2_, and TiO_2_ at 93–95 K at relatively low D_2_O exposures (below 900 L). D_2_O exposure conditions are as follows: Si wafer, 5 × 10^−6^ mbar for 180 s; Al_2_O_3_, 4 × 10^−7^ mbar for 1200 s; ZrO_2_, 2 × 10^−7^ mbar for 600 s; and TiO_2_, 2 × 10^−7^ mbar for 1800 s.

At the liquid D_2_O–air interface,^34^ three characteristic peaks appeared at 2390 (broad), 2520 (broad), and ∼2725 cm^−1^, corresponding to strongly H-bonded OD, weakly H-bonded OD, and dangling OD groups, respectively. Fig. 4 compares the ppp-SFG spectra of D_2_O adsorbed on a Si substrate and on Si-supported Al_2_O_3_, ZrO_2_, and TiO_2_ films at 93–95 K under low D_2_O exposure. It has been reported that dosing 5 L of D_2_O at 90 K initially forms an amorphous ice multilayer;^41^ upon heating to 160 K, an ordered monolayer is formed which remains stable up to 210 K.^57^ Therefore, the spectra shown in Fig. 4 originate from multilayer ice. For reference, the ice growth rate on Pt(111) has been reported to be 0.03 monolayers (ML) per second at a water pressure of 5 × 10^−8^ torr.^39^ Herein, we assumed that 1 L of ice corresponds to a water flux of 10^−6^ mbar s.

Five distinct features were observed in Fig. 4, centered at approximately 2310, 2350, 2450, 2650 and 2730 cm^−1^. Due to the presence of multiple species that may exhibit different phases, the spectra were not fitted at this stage, though. The bands are tentatively assigned to strongly hydrogen-bonded OD stretching of highly ordered water (D_2_O) molecules in ice (D_2_O–D_2_O H-bonded OD_1_), CO_2_ gas adsorption (originating from residual CO_2_ in the lab atmosphere beam path), weakly H-bonded O–D stretching of less-ordered D_2_O molecules (D_2_O–D_2_O H-bonded OD_2_), weakly H-bonded OD stretching of D_2_O interacting with the oxide film (D_2_O–oxide H-bonded OD_3_), and the “free” OD stretching of the topmost D_2_O molecules, respectively. The D_2_O–D_2_O H-bonds may arise from both interlayer and intralayer water molecules.^42^ A peak near 2730 cm^−1^ corresponding to “free” OD groups has also been reported for intact water molecules weakly bound on the terrace sites of Mn_3_O_4_(111).^58^ Due to strong interference between the non-resonant signal from the substrate (*i.e.*, Si-wafer supported oxide film) and the resonant signal from the ice layer, the spectral lineshape of the free O–D stretch appears highly asymmetric (Fano resonance, similar to ice on MgO(001)).^41^ Based on the lineshapes reported by Miranda and Shen,^59^ the non-resonant surface nonlinear susceptibility is real and has an opposite sign to the resonant contribution. In contrast, on the pure Si wafer surface, the lineshape of the free O–D mode is closer to a symmetric (*i.e.*, more conventional) profile. This suggests that the strong interference observed in our case likely originates from the metal oxide films.

On the Al_2_O_3_ surface under low exposure conditions, the absence of the H-bonded OD_1_ feature indicates that the surface is initially populated primarily by water species with a “free” (non-hydrogen-bonded) OD group, alongside water species exhibiting weak water–water and water–oxide interactions. This suggests that strong interlayer and intralayer molecular interactions among water molecules are established only at higher coverages. Consequently, we performed additional measurements at varying D_2_O exposures at 93–95 K.

#### Variation of D_2_O exposures

##### Si wafer

The H-bonded OD_3_ band (∼2650 cm^−1^) in Fig. 4, assigned to H-bonds between water and the oxide film (this species awaits confirmation by fitting the individual peaks, though), was observed on all oxide films (Al_2_O_3_, ZrO_2_, and TiO_2_). For D_2_O (10 layers) adsorption on 0.5 ML CO/Pt(111) at 140 K, four O–D features have been reported: two broad bands at 2278 and 2472 cm^−1^ assigned to hydrogen-bonded OD symmetric and asymmetric stretching modes, respectively, and two weaker bands at 2675 and 2720 cm^−1^ corresponding to O–D stretching of D_2_O interacting with CO at the D_2_O/CO interface and the “free” OD at the vacuum/ice interface.^60^ The peak at 2675 cm^−1^ is qualitatively like the OD_3_ feature observed in Fig. 4.

This assignment is supported by its absence on the Si substrate. However, it is also possible that the signal on Si is simply too weak to detect. Furthermore, because a D_2_O molecule contains two OD groups, one can act as “free” OD (at ∼3730 cm^−1^) pointing toward the vapor phase, while the other points toward the oxide surface. The latter OD group may form H-bonds either with the oxide film or with neighboring water molecules. For example, at the liquid–air interface, a broad peak at 3550 cm^−1^ has been attributed to the H-bonded O–H stretching mode with *C*_∞v_ symmetry, arising from water molecules with one “free” and one H-bonded OH group.^61^ If the OD group responsible for the OD_3_ band were primarily H-bonded to neighboring water molecules, then this feature should also appear on the Si surface. To rule out these possibilities, we measured the SFG spectra of D_2_O adsorption on the Si substrate as a function of D_2_O exposure—controlled by adjusting the D_2_O vapor pressure and/or the exposure time—corresponding to progressively thicker ice layers.

**Fig. 5:**
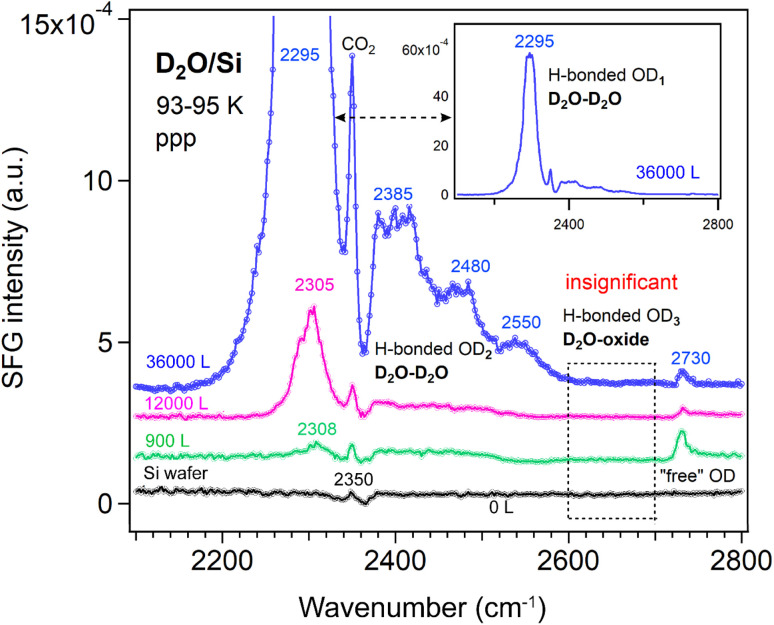
ppp-SFG spectra of D_2_O adsorbed on a Si wafer with increasing D_2_O exposure at 93–95 K. The inset shows the full-scale spectrum obtained after exposure to 3 × 10^−4^ mbar D_2_O for 2 min at 93–95 K. D_2_O exposure conditions are as follows: black, no; green, 5 × 10^−6^ mbar for 180 s; pink, 1 × 10^−4^ mbar for 120 s; and blue, 3 × 10^−4^ mbar for 120 s.

However, the exposure-dependence of SFG spectra clearly rules out such possibilities. As shown in Fig. 5, no signal appears in the 2600–2700 cm^−1^ region. Therefore, the OD_3_ stretching mode in Fig. 4 most probably originates from hydrogen bonding interactions involving water molecules (*e.g.*, DAA, DA) with one “free” OD group or from species (*e.g.*, DDA) in which both OD groups are bonded directly to the oxide surface. This band also may be assigned to dissociated water species, as their stretching frequency is lower than that of free O–D in molecularly adsorbed D_2_O.^62^

Additionally, as D_2_O exposure increases on the Si wafer, the intensity of the “free” OD signal decreases, while the weakly H-bonded OD_2_ and strongly H-bonded OD_1_ peaks increase. The weak OD_2_ peak, however, remains several times weaker than the strong OD_1_ peak.

Similarly, D_2_O adsorption on the Al_2_O_3_, ZrO_2_, and TiO_2_ ALD films at 93–95 K was systematically investigated as a function of D_2_O exposure. All SFG spectra were recorded in the 2150–2800 cm^−1^ range. To clearly illustrate the spectral evolution, the spectra corresponding to the H-bonded OD_2_, H-bonded OD_3_, and “free” OD modes are presented separately, each with an appropriately scaled *y*-axis.

##### Al_2_O_3_ film

**Fig. 6:**
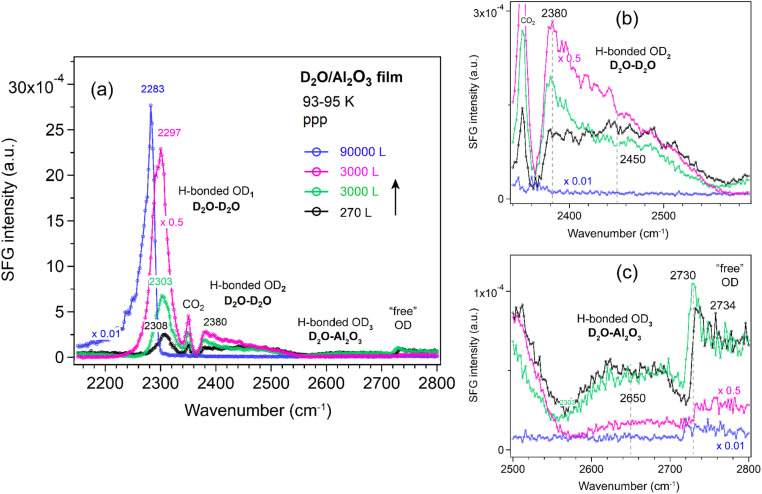
(a) ppp-SFG spectra in the O–D stretching region of D_2_O adsorbed on an ALD-grown Al_2_O_3_ film on Si wafer with increasing D_2_O exposure at 93–95 K. For clarity, ppp spectra with appropriately adjusted *y*-axis scales are shown in (b) for the OD_2_ region (2340–2580 cm^−1^) and in (c) for the OD_3_ and “free” OH regions (2500–2800 cm^−1^). D_2_O exposure conditions are as follows: black, 1.5 × 10^−6^ mbar for 180 s; green, 5 × 10^−5^ mbar for 60 s; pink, 1 × 10^−4^ mbar for 30 s; and blue, 2.5 × 10^−5^ mbar for 3600 s.

Fig. 6 shows the ppp-SFG spectra of D_2_O adsorption on the Al_2_O_3_ film. The five peaks (also shown in Fig. 4) vary differently with increasing ice layer thickness. As the D_2_O exposure increases, the OD_1_ stretching band shifts to lower wavenumbers (from 2308 to 2283 cm^−1^) and increases in intensity (Fig. 6a). This behavior can be attributed not only to stronger intermolecular interactions (or transition dipole coupling) among water molecules but also to an increased population of molecules adopting an ordered structure. On Pt(111) single crystal surfaces, the strong enhancement of the O–H stretching resonance with increasing ice thickness arises from surface-induced polar ordering, generated by polar anchoring of the first ice monolayer on Pt.^39^

In contrast, the weakly bonded OD_2_ stretching band (Fig. 6b) initially increases and then decreases in intensity. At low exposure (black curve), the OD_2_ band is broad (2450 cm^−1^), but it becomes narrower and shifts to lower wavenumbers (2380 cm^−1^) as the exposure increases (green and pink curves).

**Fig. 7:**
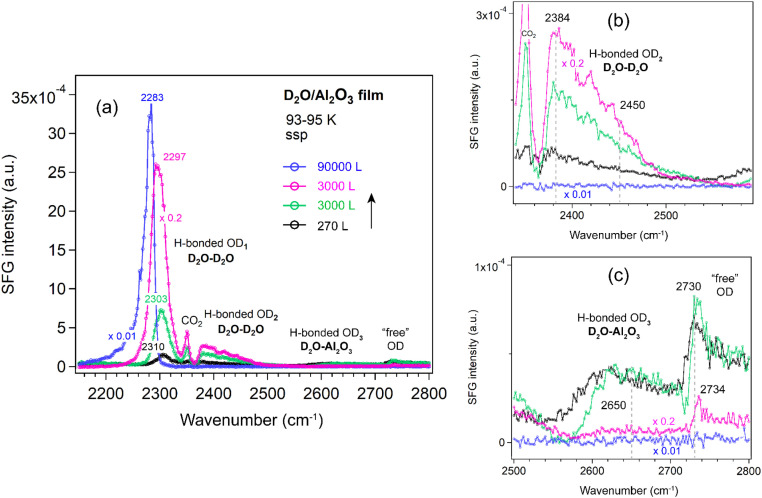
(a) ssp-SFG spectra in the O–D stretching region of D_2_O adsorbed on an ALD-grown Al_2_O_3_ film on Si wafer with increasing D_2_O exposure at 93–95 K. For clarity, ssp spectra with appropriately adjusted *y*-axis scales are shown in (b) for the OD_2_ region (2340–2580 cm^−1^) and in (c) for the OD_3_ and “free” OH regions (2500–2800 cm^−1^). D_2_O exposure conditions are as follows: black, 1.5 × 10^−6^ mbar for 180 s; green, 5 × 10^−5^ mbar for 60 s; pink, 1 × 10^−4^ mbar for 30 s; and blue, 2.5 × 10^−5^ mbar for 3600 s.

The weakly H-bonded OD_3_ stretching and “free” OD bands show trends similar to OD_2_, but they vanish at earlier stages—disappearing at an exposure of 1 × 10^−4^ mbar for 30 s (Fig. 6c). These observations are further supported by the SFG spectra collected using the ssp polarization combination (Fig. 7a–c). According to the SFG selection rules,^63^ since at 270 L the OD_2_ peak at 2450 cm^−1^ is stronger in the ppp spectrum (Fig. 6b) than in the ssp (Fig. 7b) spectrum, whereas the 2380 cm^−1^ peak is stronger in ssp (Fig. 7b) than in ppp (Fig. 6b), the former can be assigned to the O–D asymmetric stretch, and the latter to the symmetric stretch of weakly bonded water. When the surface is exposed to 2.5 × 10^−5^ mbar D_2_O for one hour (blue curve), the OD_1_ signal increases several times, while all other bands disappear completely.

The hydrogen-bond network in D_2_O can be viewed analogously to that in H_2_O. ppp-SFG studies of H_2_O-ice (−17 °C) and liquid water (23 °C) adjacent to a sapphire (Al_2_O_3_) prism have been reported.^31^ Compared with liquid water, the strongly H-bonded O–H peak is red-shifted from ∼3200 to ∼3150 cm^−1^ in ice, indicating stronger intermolecular hydrogen bonding. In contrast, the “free” O–H mode is blue-shifted from ∼3700 to ∼3740 cm^−1^ and decreases in intensity. Notably, no distinct weakly bonded O–H features associated with H_2_O–H_2_O and H_2_O–Al_2_O_3_ interactions were observed.^31^ The absence of these signals is probably due to the reduced structural ordering of water molecules at 256 K compared with the highly ordered structure at 95 K (Fig. 6).

The strongest peak at 3098 cm^−1^, assigned to water molecules forming bilayer-stitching hydrogen bonds, contained a substantial quadrupole bulk contribution that produces a 90° phase shift relative to a purely interfacial mode.^64^ A new O–H stretching band of H_2_O-ice at 3530 cm^−1^ has been observed by heterodyne-detected (HD)-SFG and arises from a combination of the asymmetric O–H stretch of fully coordinated DDAA molecules and the symmetric O–H stretch of DDA water molecules with opposite phase.^64^

MD and *ab initio* studies revealed that the structural influence of graphene on water is extremely local: strong ordering is observed only in the first water layer, while subsequent layers exhibit bulk-like behavior. Stratification does not persist beyond ∼5 Å from the graphene surface.^65^ Thus, at high water exposures (Fig. 6 and 7), the SFG signal primarily reflects the ice–vapor interface rather than the ice–oxide interface, consistent with the disappearance of the OD_3_ peak (associated with hydrogen bonds between water and the oxide film).

For ice on metal single crystal surfaces, the behavior is different. On Pt(111),^39^ as the ice film thickness increased from 1.2 to 26.4 ML, the H-bonded O–H peak (∼3100 cm^−1^) intensities were dramatically enhanced and blue-shifted, in contrast to the red-shift behavior observed in Fig. 6.

The Imχ^(2)^ SFG spectrum of the H-bonded O–H (or O–D) stretching region of H_2_O (D_2_O) ice film exhibits multiple peaks with exclusively negative signs, indicating net-H-down (or net-D-down) ferroelectric orientational ordering in which protons (or deuterons) preferentially point toward the Pt substrate.^66^ The orientation of the interfacial water plays a key role in ice structure: freezing water next to a positively charged sapphire surface yields a stronger ice signal than liquid water, whereas freezing near a negatively charged mica surface produces proton-disordered ice, causing strong attenuation of the SFG signal.^67^

Because the ssp spectra of the D_2_O on Al_2_O_3_ film (Fig. 7) showed no additional features or significant enhancements compared to the ppp spectra (Fig. 6), all spectra of the other oxides (ZrO_2_, TiO_2_, and CoO) were measured only in the ppp polarization combination.

##### ZrO_2_ film

**Fig. 8:**
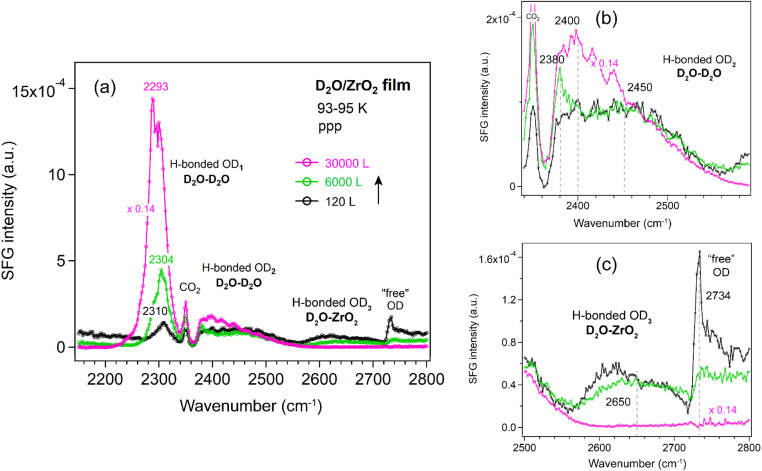
(a) ppp-SFG spectra in the O–D stretching region of D_2_O adsorbed on an ALD-grown ZrO_2_ film on Si wafer with increasing D_2_O exposure at 93–95 K. For clarity, ppp spectra with appropriately adjusted *y*-axis scales are shown for (b) the OD_2_ region (2340–2580 cm^−1^) and (c) the OD_3_ and “free” OH regions (2500–2800 cm^−1^). D_2_O exposure conditions are as follows: black, 2 × 10^−7^ mbar for 600 s; green, 1 × 10^−5^ mbar for 600 s; and pink, 5 × 10^−5^ mbar for 600 s.

Similar trends as for Al_2_O_3_ were observed for the ZrO_2_ film (Fig. 8). The OD_1_ band increased in intensity and shifted to lower wavenumbers (from 2310 to 2293 cm^−1^). Overall, a decrease in the OD_3_ and “free” OD bands was observed with increasing D_2_O exposure, consistent with the behavior seen on Si (Fig. 5) and Al_2_O_3_ (Fig. 6) surfaces.

##### TiO_2_ film

**Fig. 9:**
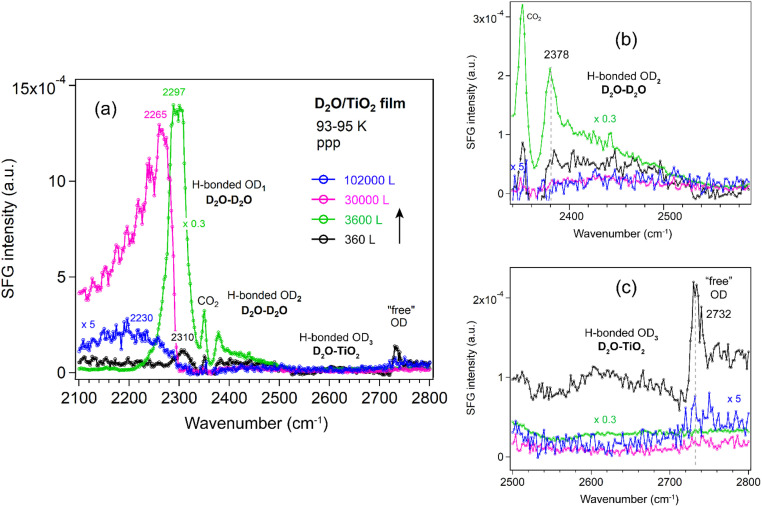
(a) ppp-SFG spectra in the O–D stretching region of D_2_O adsorbed on an ALD-grown TiO_2_ film on Si wafer with increasing D_2_O exposure at 93–95 K. For clarity, ppp spectra with appropriately adjusted *y*-axis scales are shown for (b) the OD_2_ region (2340–2580 cm^−1^) and (c) the OD_3_ and “free” OH regions (2500–2800 cm^−1^). D_2_O exposure conditions are as follows: black, 2 × 10^−7^ mbar for 1800 s; green, 1 × 10^−5^ mbar for 360 s; pink, 1 × 10^−4^ mbar for 300 s; and blue, 1 × 10^−4^ mbar for 1020 s.

For TiO_2_ films (Fig. 9), the OD_1_ band initially behaves similarly to that on Al_2_O_3_ and ZrO_2_, showing an increase in intensity and a shift from 2310 to 2293 cm^−1^ at low D_2_O exposure. At higher exposure (1 × 10^−4^ mbar), prolonged dosing leads to a decrease in intensity and a pronounced shift to lower wavenumbers (2265–2230 cm^−1^). This behavior is consistent with earlier reports that the crystallization rate of amorphous solid water decreases sharply with increasing film thickness, likely to be crystallization-induced cracking.^68^ The observed OD_1_ decrease on TiO_2_ is therefore attributed to film cracking under high water vapor pressures. When the sample is cooled from the bottom using liquid N_2_, crystallization of the thin film proceeds more slowly than ice sublimation. In addition, SFG studies of D_2_O at the solid–liquid interface of TiO_2_ films with thickness of 85 and 150 nm have been reported;^69^ however, no peak was observed in the 2600–2700 cm^−1^ region.

It was reported that the variation in the H_2_O–metal binding energy varies little from metal to metal.^28^ As Al_2_O_3_ and ZrO_2_ films were only exposed to relatively lower vapor pressures (<1 × 10^−4^ mbar), a similar decrease in the OD_1_ band would also be expected if they were exposed to higher vapor pressures for longer durations. Therefore, there is no significant difference in the overall water structure among the Al_2_O_3_, ZrO_2_, and TiO_2_ films.

##### CoO film

**Fig. 10:**
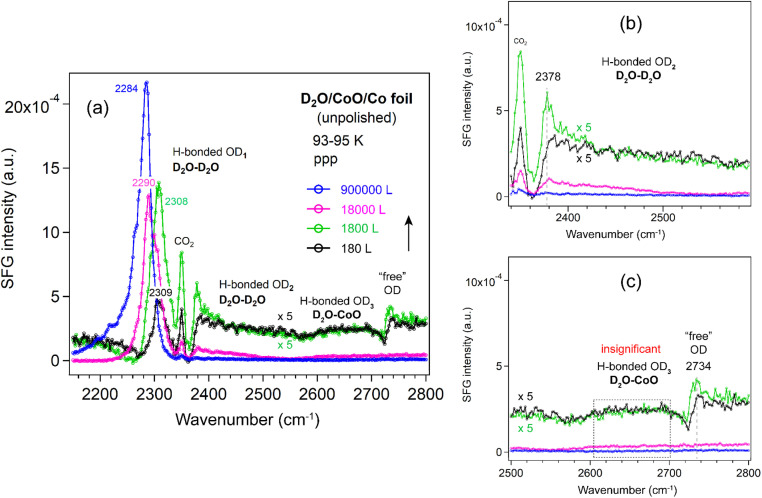
(a) ppp-SFG spectra in the O–D stretching region of D_2_O adsorbed on CoO film/unpolished Co foil with increasing D_2_O exposure at 93–95 K. For clarity, ppp spectra with appropriately adjusted *y*-axis scales are shown in (b) for the OD_2_ region (2340–2580 cm^−1^) and in (c) for the OD_3_ and “free” OH regions (2500–2800 cm^−1^). D_2_O exposure conditions are as follows: black, 1 × 10^−6^ mbar for 180 s; green, 1 × 10^−5^ mbar for 180 s; pink, 1 × 10^−4^ mbar for 180 s; and blue, 5 × 10^−4^ mbar for 1800 s.

To complement our observations on the three ALD-grown films, we also measured the SFG spectra of D_2_O adsorption on a CoO film under similar conditions (Fig. 10). The CoO was prepared by oxidizing an unpolished Co foil in 10^−6^ mbar O_2_ at 873 K for 5 h (similar to ref. 70). The subsequent LEIS spectrum (Fig. S4) showed two features assigned solely to Co and O surface species based on a LEIS calculator.^71^ The low background intensity below 500 eV further confirmed a clean surface without impurities such as carbon.^72^ XPS analysis of the Co 2p region (Fig. S5) revealed no metallic Co signals and showed features characteristic of CoO, consistent with the work of Biesinger *et al.*^73^ Deconvolution of the Co 2p spectrum showed two contributions associated with Co^2+^ at 779.9 eV and 782.1 eV (Co 2p_3/2_), accompanied by two shake-up satellites and a spin–orbit splitting of 15.9 eV (Fig. S5), in agreement with literature.^73–75^ In the O 1s region (Fig. S6), the main peak at 529.8 eV was assigned to lattice oxygen in CoO, while a weak shoulder at around 531.1 eV was attributed to adsorbed oxygen species such as OH groups.^73,75,76^ Furthermore, the Co : O stoichiometry determined from the relative XPS peak intensities was approximately 1 : 1, confirming the formation of a CoO film on the Co foil.

The CoO film was then transferred in air for SFG measurements. To remove potential contaminants introduced during air exposure, the CoO film was oxidized in 1 × 10^−6^ mbar O_2_ at 573 K for 60 min, following a similar procedure used for the ALD-grown oxides. After pretreatment, the spectrum of the CoO film was very similar to that of a Si-wafer (Fig. S7) and the ALD-grown oxide films (Fig. 2), indicating that there were no significant differences in the non-resonant SFG signals.

After growing ice on the CoO film, the OD_1_, OD_2_ and “free” OD bands (Fig. 10) exhibited trends similar to those observed on Al_2_O_3_ and ZrO_2_ films. Because the Co foil was unpolished, the resulting CoO film was grown on a substrate with sub-micrometer roughness (Fig. S8). Consequently, due to strong signal scattering from the rough surface, the “free” OD peak was at least 5 times weaker than on the flat ALD-grown films, and the OD_3_ band (water–oxide) could not be detected. Furthermore, even after exposure to 5 × 10^−4^ mbar D_2_O for 30 min, no decrease in the OD_1_ band (2284 cm^−1^) is observed, in contrast to the TiO_2_ film, where the OD_1_ band shifts to 2230 cm^−1^ at 1 × 10^−4^ mbar after 17 min. Given the >50 cm^−1^ red shift of the OD_1_ band on TiO_2_, these results clearly indicate that the water structure on the TiO_2_ film, especially at higher vapor pressures, behaves differently from that on Al_2_O_3_, ZrO_2_, and CoO films, regardless of whether the oxide is ALD-grown or formed by direct oxidation of a metal foil. For completeness we mention that roughness on the nanometer-scale may enhance SFG signals, such as that of CO adsorbed on rough Ir surfaces (created by ion-sputtering), as compared to smooth Ir(111).^77^ The enhancement is attributed to localized surface plasmon resonances (LSPR). Similarly, CO on 45 nm Pt nanoparticles yielded much a stronger signal than on smooth Pt films.^78^

#### SFG spectra of D_2_O ice adsorbed on a Si substrate and three oxide films in the O–H stretching region (3000–3800 cm^−1^)

**Fig. 11:**
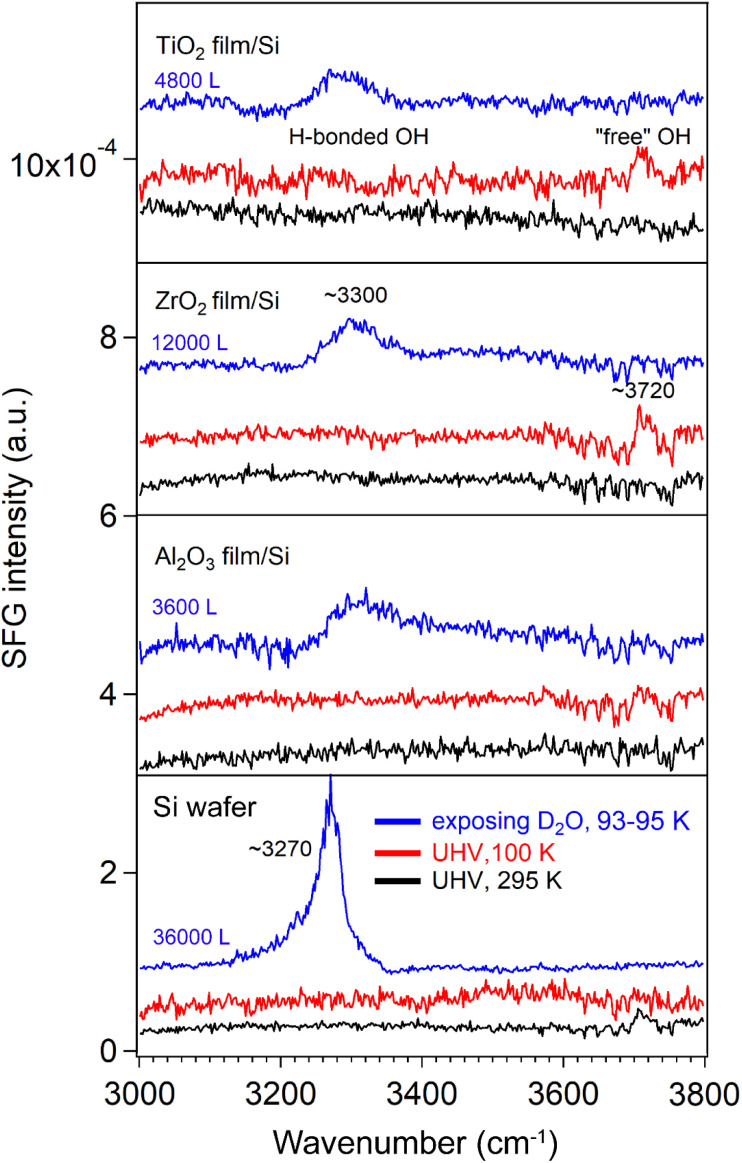
ppp-SFG spectra in the O–H stretching region for bare Si substrate and ALD-films of Al_2_O_3_, ZrO_2_, and TiO_2_ surfaces. D_2_O exposure conditions are as follows: bare Si, 3 × 10^−4^ mbar for 120 s; Al_2_O_3_ film/Si, 2 × 10^−6^ mbar for 1800 s; ZrO_2_ film/Si, 2 × 10^−5^ mbar for 600 s; and TiO_2_ film/Si, 1 × 10^−5^ mbar for 480 s.

Under ambient conditions, most metal oxide surfaces are decorated with hydroxyl groups, a consequence of water dissociative chemisorption.^79^ The SFG signal from surface hydroxyl groups can be quenched by the addition of methanol.^80^ When exposed to an aqueous environment, pristine minerals such as SiO_2_,^81^ Al_2_O_3_,^82^ and TiO_2_^83^ can hydroxylate to form terminal hydroxyl (–OH) groups. However, our results showed that no OH groups were detectable at room temperature (295 K) (Fig. 11).

After dosing with high-pressure D_2_O, a weak and broad hydrogen bonded O–H stretching band^84^ centered at 3300 cm^−1^ was observed on all ALD-grown oxide films, likely to be trace H_2_O introduced to the chamber *via* the gas line. This peak shifted to 3270 cm^−1^ on the Si substrate at higher D_2_O exposure. Furthermore, a similar red shift from the hydrogen-bonded O–H band to the OD_1_ band was observed, accompanied by the disappearance of the “free” OH signal. These findings are consistent with Fig. 6–10 and agree with previous studies reporting that at 80 K, no free OH bond was present in the H-down bilayer structure.^81^ In addition, the relatively weak H-bonded OH and “free” OH signals in Fig. 11 were not observed on the rough CoO/Co foil surfaces (Fig. S9), also due to signal scattering from rough surfaces.

## Conclusions and outlook

SFG spectra of D_2_O adsorption (formation of ice or amorphous solid water) on ALD-grown Al_2_O_3_, ZrO_2_, and TiO_2_ films at 93–95 K have been investigated. It was revealed that the interaction between ice and the oxide surfaces was relatively weak, primarily exhibiting a broad O–D stretching feature near 2650 cm^−1^ (OD_3_ band), with minimal dependence on oxide composition. This feature was observed for the first time and remained detectable up to exposures of 3000 L on Al_2_O_3_, 6000 L on ZrO_2_, and 360 L on TiO_2_. In contrast, it was not observable on the CoO film supported on a rough Co foil because the signal was too weak, probably due to signal scattering. Upon further increase in D_2_O exposure (>10^4^ L), spectral contributions from the ice–oxide interface were progressively overshadowed by those from the ice–vapor interface (OD_1_ and OD_2_ bands). At sufficiently high exposures, the disappearance of the “free” O–D stretch indicated that all interfacial molecules became hydrogen-bonded, consistent with the formation of an H-down bilayer structure.^81^

At the ice–vapor interface (≥30 000 L), among the oxides studied, TiO_2_ exhibits notably distinct behavior: the strongly hydrogen-bonded OD_1_ mode undergoes an anomalous red-shift to 2230 cm^−1^ accompanied by significant intensity loss, indicating the formation of unique water structures on this surface. No significant differences in water structure were observed on the ALD-Al_2_O_3_, -ZrO_2_, and CoO films/Co foil, aside from an approximately fivefold reduction in intensity on CoO, which was attributed to scattering losses caused by the sub-micron scale rough CoO film/Co foil surface. In contrast, for CO adsorption on rough Ir surfaces (on the nanometer scale), as compared to smooth Ir(111), an eightfold enhancement in SFG intensity has been observed due to a light-induced excitation of localized surface plasmon resonances.^77^

These findings underscore the importance of oxide surface structure in governing water adsorption behavior. Previous studies have demonstrated that water dissociation is enhanced on ultrathin MgO films compared to bulk-like MgO(001),^62^ motivating further investigation of thickness-dependent SFG responses in ALD-grown Al_2_O_3_, ZrO_2_, and TiO_2_ films. At the liquid D_2_O–TiO_2_ interface (with TiO_2_ deposited by ALD on a CaF_2_ window), SFG spectra showed that the differences between samples with 85 *vs.* 150 nm TiO_2_ film were independent of film thickness.^69^ Extending similar investigations to ALD oxides may yield valuable insights for designing surfaces with reduced ice adhesion.

## Experimental methods

### Sample preparation

#### ALD-grown oxide films

The Siegert Wafer SiO_2_/Si wafer (525 µm N-type, phosphorus-doped, <100>, 1–5 Ω) (7 × 7 mm^2^) was used as the substrate for atomic layer deposition (ALD). Before deposition, the Si wafers were sonicated in acetone and methanol to remove glue residues and then dried in N_2_. Al_2_O_3_ and TiO_2_ thin films were deposited onto the Si substrate using thermal-mode ALD in an R-200 standard reactor (Picosun, Finland). ZrO_2_ films were grown using a Gemstar thermal ALD system (Arradiance, LLC).

Al_2_O_3_ films were prepared using alternating exposures of trimethylaluminum (TMA, EpiValence) (Al precursor) and H_2_O (EpiValence). Each ALD cycle consisted of a pulse–purge sequence: 0.1 s TMA pulse, 5 s N_2_ purge, 0.1 s H_2_O pulse, and 8 s N_2_ purge. TMA was stored in a stainless-steel bubbler maintained at 22 °C. The reaction chamber temperature was set to 150 °C and the chamber pressure to 9 hPa. In all cases, the H_2_O reservoir was kept at 22 °C, and the ultrahigh purity nitrogen (Messer Technogas, 99.999%) was used as the carrier and purge gas. A total of 40 ALD cycles were performed.

ZrO_2_ films were deposited using tetrakis(dimethylamino)zirconium(iv) (TDMAZ, Sigma-Aldrich) and deionized H_2_O. The manifolds and chamber temperatures were maintained at 140 °C. N_2_ (99.999%) was used as carrier gas at a flow of 50 SCCM. The TDMAZ bubbler was kept at 75 °C and the H_2_O bubbler at room temperature. The TDMAZ and H_2_O pulse/purge times were 50 ms/12 s and 100 ms/12 s per ALD cycle, respectively. A total of 35 ALD cycles were performed.

TiO_2_ films were deposited using tetrakis(dimethylamido)titanium(iv) (TDMATi from Strem Chemicals) and H_2_O (EpiValence). The TDMATi was evaporated at 85 °C. The substrate temperature was 150 °C and the chamber pressure was 1 kPa during deposition. One TiO_2_ ALD cycle consisted of a 1.6 s TDMATi pulse, 6 s N_2_ purge, 0.1 s H_2_O pulse, and 8 s N_2_ purge. A total of 83 ALD cycles were performed.

#### CoO oxide film

A polycrystalline cobalt foil (1 × 1 cm^2^, 99.9% purity, MaTecK GmbH) was cleaned according to the procedure described by Wu *et al.*^85^ to obtain a contaminant-free Co surface. The effectiveness of the cleaning process was verified by XPS and LEIS. The cleaned foil was subsequently oxidized by annealing in an oxygen atmosphere (*p*_O_2__ = 1 × 10^−6^ mbar) at 300 °C for 5 h, until the absence of metallic Co signals in the XPS spectrum confirmed complete oxidation of the near-surface region of the polycrystalline Co foil. After oxidation, a LEIS spectrum was recorded.

#### D_2_O ice

D_2_O ice was prepared by water vapor deposition. D_2_O was exposed to the chamber after a freeze–thaw cycle.

### Ellipsometry

The oxide thickness was determined by spectroscopic ellipsometry using an EP4 imaging ellipsometer (Accurion GmbH) equipped with a 10× objective and a 50° angle of incidence, over the spectral range 360–1000 nm (filter-wheel configuration with 45 wavelengths).

### X-ray photoelectron spectroscopy (XPS) and low-energy ion scattering (LEIS)

XPS and LEIS were conducted in a dedicated ultra-high-vacuum chamber (UHV 1) (35 L) with a base pressure ≤5 × 10^−10^ mbar. As described in ref. 86 and 87, the system was equipped with a high-intensity, non-monochromatic Al/Mg dual-anode X-ray source (XR50, SPECS GmbH) and a hemispherical energy analyzer (Phoibos 100©) with a multichannel plate detector. For ALD-grown oxides, Al Kα radiation (1486.61 eV) was used for the acquisition of XPS spectra. For CoO film, Mg Kα radiation (1253.6 eV) was used with an emission angle of 0° and an analyzer pass energy of 20 eV. All XPS spectra were acquired at room temperature. Before XPS, all samples were thoroughly cleaned by a cycle of oxidation (1 × 10^−6^ mbar O_2_, 923 K, 30 min) and reduction (1 × 10^−6^ mbar H_2_, 923 K, 30 min).

LEIS measurements were performed using a SPECS IQE 12/38© ion source operated with He^+^ ions at a kinetic energy of 1 keV, a helium backpressure of 2 × 10^−7^ mbar, and a scattering angle of 135°.

### SFG spectroscopy

The SFG cell can be operated from 2.5 × 10^−8^ mbar to 1 bar pressure and at 100–800 K.^77,88,89^ SFG measurements were performed using a 20 ps mode-locked Nd:YAG laser system (EKSPLA, PL2241) with a fundamental radiation of 1064 nm (30 mJ per pulse, 50 Hz repetition rate).^88^ A tunable mid-infrared beam (with the photon energy ωIR) and a visible beam with a fixed wavelength of 532 nm were directed in a co-propagation geometry toward the surface, with incidence angles of 55° and 58.5° with respect to the surface normal, respectively. The pulse energy was 90–130 µJ for infrared between 2150 and 3800 cm^−1^ and 30 ± 5 µJ for visible. The SFG signal was collected/detected in the reflection direction with a photomultiplier tube (PMT). The polarization of IR was kept as P and that of visible and SFG signal was switched between P and S using a Glan–Taylor prism and a half-wave plate. All spectra were normalized by the energy of visible and IR laser pulses.

Before SFG measurements (in UHV 2), all oxide films were oxidized in 1 × 10^−5^ mbar O_2_ at 600 K for 60 min. If SFG measurements could not be finished on the same day, the next day samples were only annealed at 423 K for 30 min under UHV only to remove adsorbed H_2_O (traces in the UHV chamber).

## Author contributions

X. L. carried out the original draft writing, the conception and design of this study, and the SFG experiments and data acquisition. S. G., T. H., Š. V., and G. R. participated in manuscript preparation. M.-H. J. and Y. L. prepared ALD-ZrO_2_ films and M. Z. prepared ALD-Al_2_O_3_ and TiO_2_ films. S. G. performed the LEIS + XPS measurements of the CoO films. T. H. performed the XPS measurements for all ALD films. M.-H. J. and M. J. performed ellipsometry on ALD films. J. E. O. participated in the XPS and SFG measurements. G. R. contributed to the conception and design of this study and to writing – review and editing. X. L., Š. V. and G. R. contributed to funding acquisition.

## Conflicts of interest

There are no conflicts of interest to declare.

## Supplementary Material

FD-265-D5FD00152H-s001

## Data Availability

The data supporting this article have been included as part of the supplementary information (SI). Supplementary information: XPS data fitting information, Fig. S1, S5 and S6 are XPS spectra; Fig. S4 is a LEIS spectrum; Fig. S2, S3, S7, and S9 are SFG spectra and Fig. S8 shows photographs of samples. See DOI: https://doi.org/10.1039/d5fd00152h.
