# Supplementary material for: Probing the structure of D2O ice layers on ALD-grown ZrO2, Al2O3 and TiO2 thin films by sum frequency generation (SFG) spectroscopy
Source: Faraday Discuss. 2025 Dec 16. Online ahead of print. doi: 10.1039/d5fd00152h (PMC13122299; doi:10.1039/d5fd00152h)
Supplement: FD-OLF-D5FD00152H-s001 [file FD-OLF-D5FD00152H-s001.pdf]

This journal is © The Royal Society of Chemistry 2025

Supplementary Information

Probing the Structure of D_2O Ice Layers on ALD-Grown ZrO_2, Al_2O_3 and TiO_2 Thin Films by Sum Frequency Generation (SFG) Spectroscopy

Xia Li[1*], Susanne Gross[1], Thomas Haunold[1], Moon-Hyung Jang[2], Marketa Zukalova[3], Martin Jindra[3], Joanna E. Olszówka[4], Yu Lei[2], Štefan Vajda[4], and Günther Rupprechter[1*]

1. Institute of Materials Chemistry, TU Wien, Vienna 1060, Austria.
2. Department of Chemical and Materials Engineering, University of Alabama in Huntsville, Huntsville, AL, 35899, USA.
3. Department of Electrochemical Materials, J. Heyrovsky Institute of Physical Chemistry, Dolejškova 2155/3, 182 00 Prague 8, Czech Republic.
4. Department of Nanocatalysis, J. Heyrovsky Institute of Physical Chemistry, Prague, Czech Republic.

XPS of ALD films: During XPS fitting, the spin-orbit coupling was neglected for Si 2p and Al 2p due to the low doublet separation energies of only 0.6[1] and 0.4 eV[2], respectively, as these are below the spectral resolution of the analyzer (approximately 0.6-0.9 eV, depending on the full width at half maximum, FWHM, and signal-to-noise ratio). For peak fitting of Ti 2p and Zr 3d regions, doublet separation energies of 5.7 and 2.4 eV were constraint, according to the NIST XPS database. In case of Zr 3d, a peak area ratio of $3d_{5/2}$:$3d_{3/2}$ = 3:2 was employed, whereas the Ti 2p peak area ratio was left unconstraint due to Coster-Kronig broadening of Ti $2p_{1/2}$.

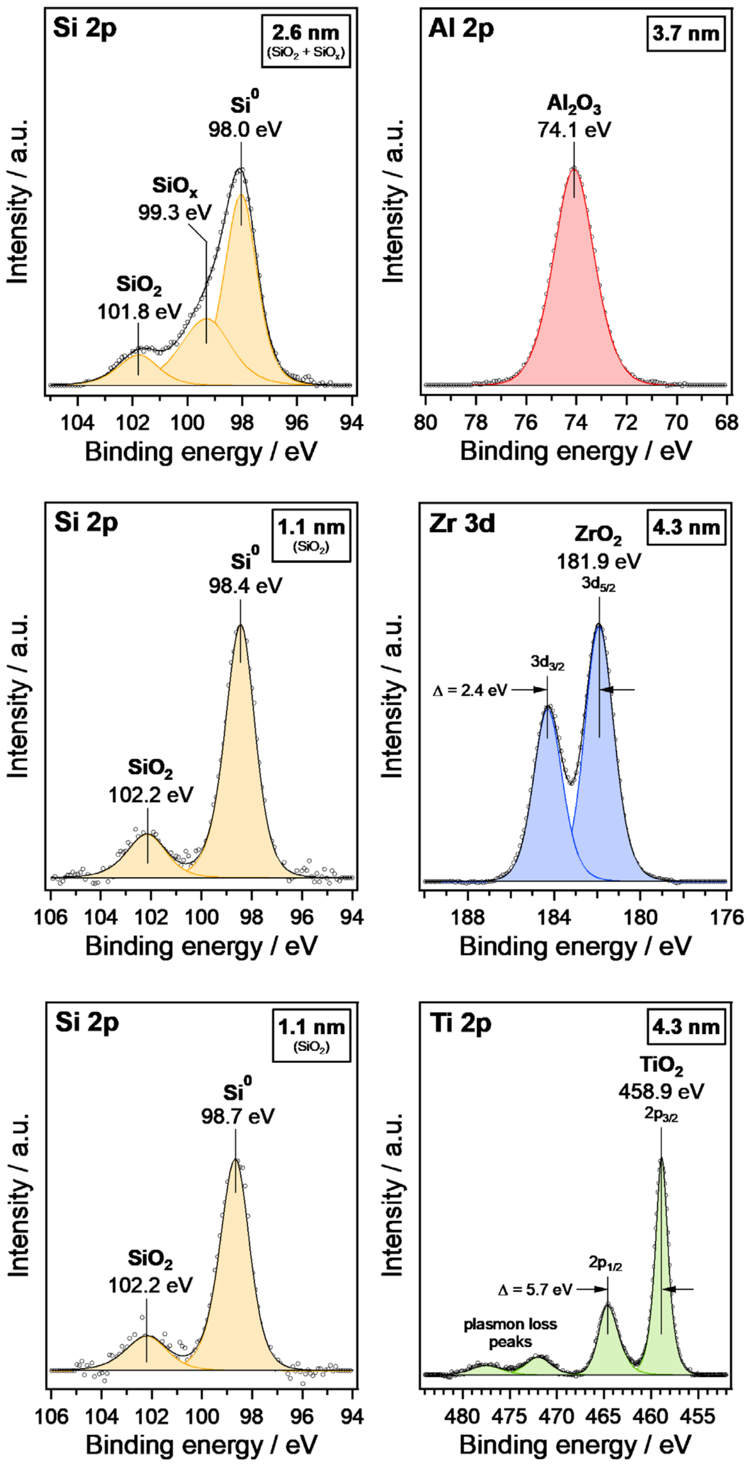

Figure S1 XPS spectra of ALD-grown Al_2O_3, ZrO_2, and TiO_2 films deposited on a Si wafer after pretreatment with oxidation ($1x10^{-6}$ mbar O_2, 923 K, 30 min) and reduction ($1x10^{-6}$ mbar H_2, 923 K, 30 min).

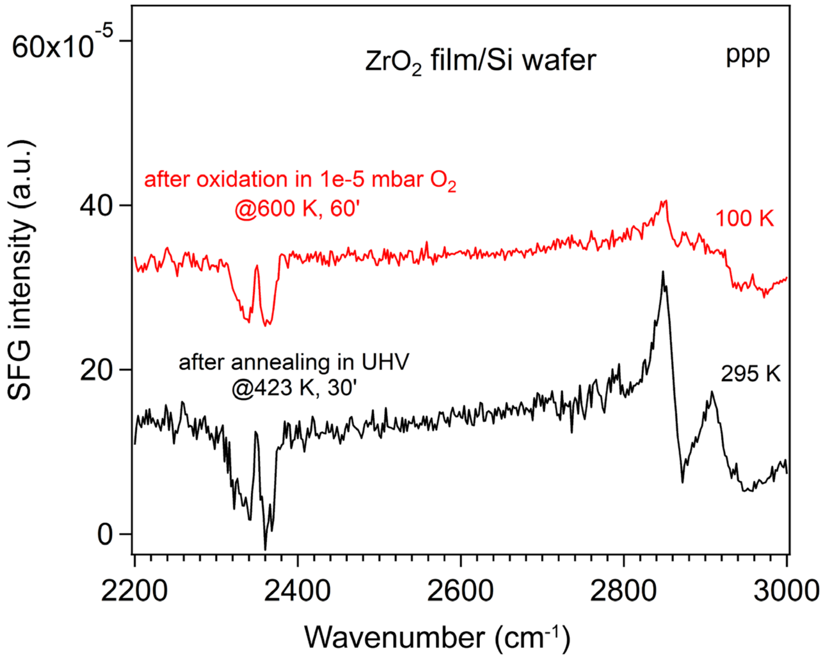

Figure S2 Comparison of ppp-SFG spectra of ZrO_2 film/Si with annealing at 423 K in UHV and at 600 K in $1x10^{-5}$ mbar O_2.

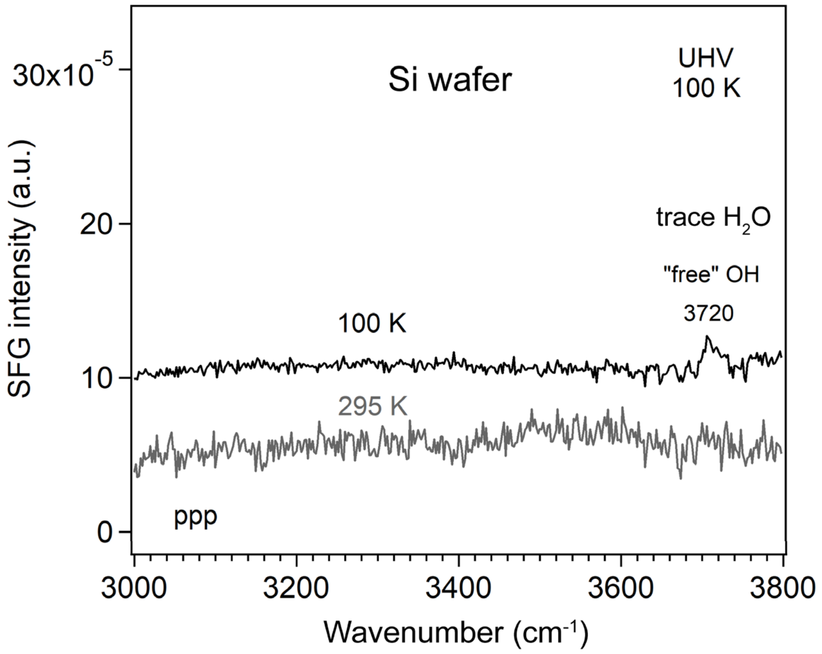

Figure S3 ppp-SFG spectra of blank Si at 100 and 295 K under UHV in the range of 3000-3800 cm^{-1}.

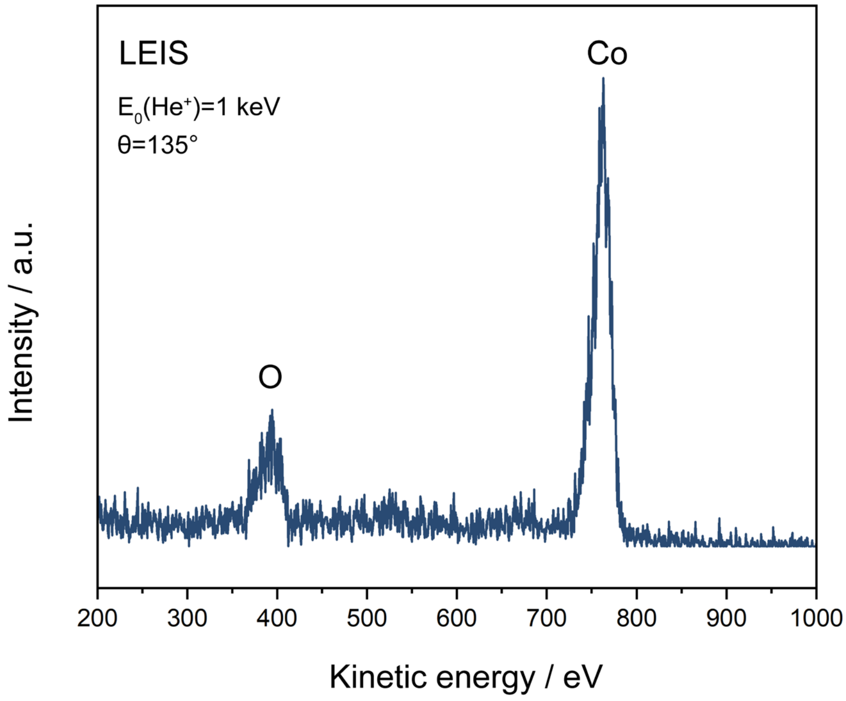

Figure S4 LEIS spectrum of the oxidized polycrystalline Co foil.

XPS Spectra of the oxidized Co foil were calibrated to the O 1s binding energy of 529.79 eV, following the reference values reported by Biesinger et al.[3] for CoO, due to the absence of adventitious carbon and the lack of a measurable Co Fermi edge caused by the oxide layer thickness. Quantification was performed using the Co 2p and O 1s regions after applying a Shirley-type and linear background subtraction, respectively.

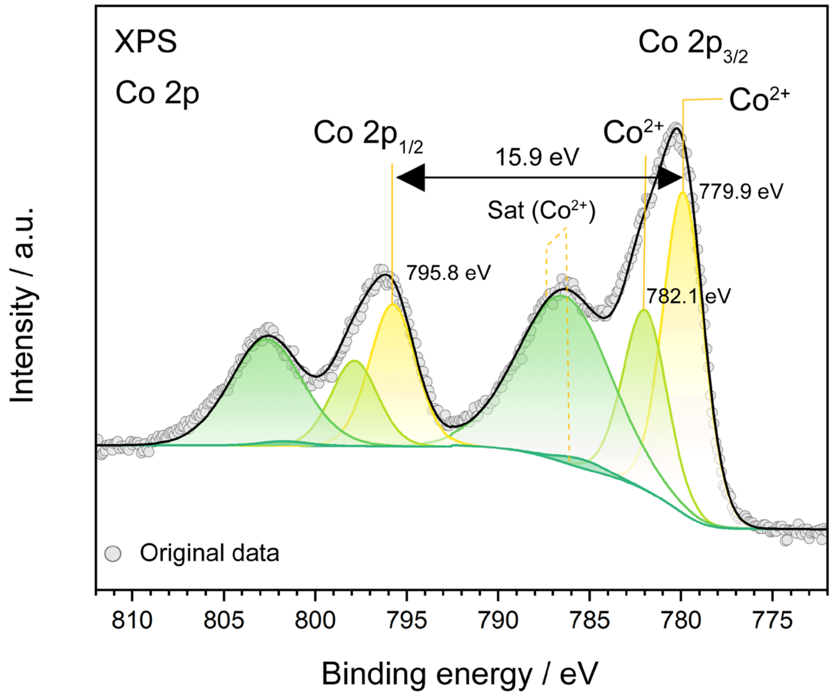

Figure S5 Co 2p-XPS spectrum of the oxidized polycrystalline Co foil.

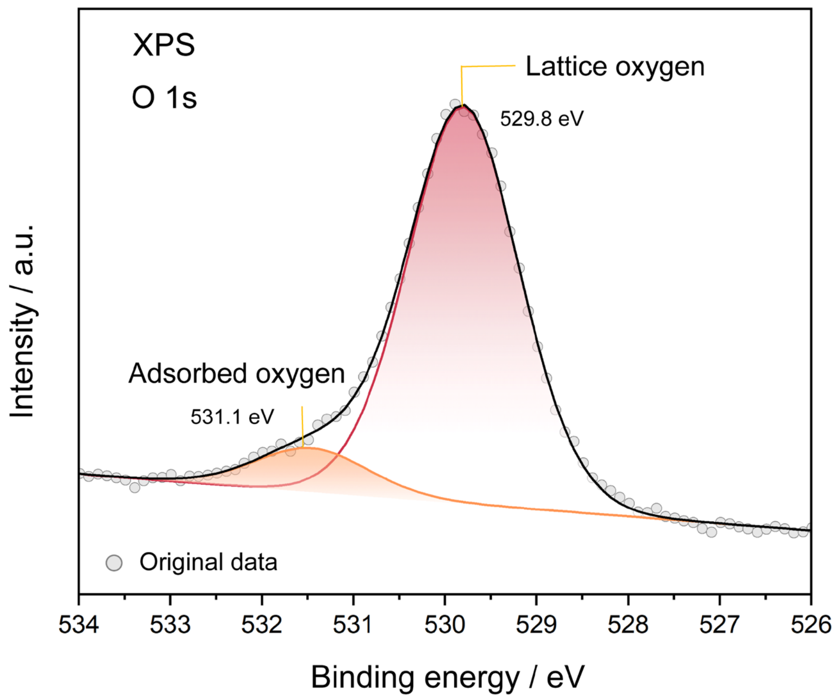

Figure S6 Co 1s-XPS spectrum of the oxidized polycrystalline Co foil.

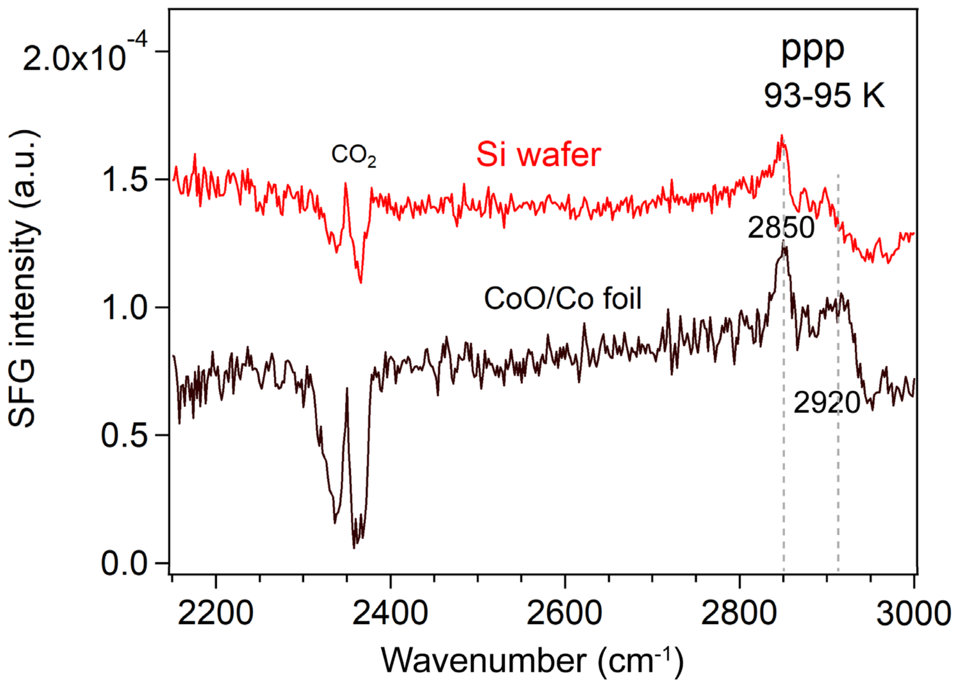

Figure S7 Comparison of ppp spectra of pure CoO film-Co foil and Si water in the range of 2150-2800 cm^{-1} acquired at 93-95 K.

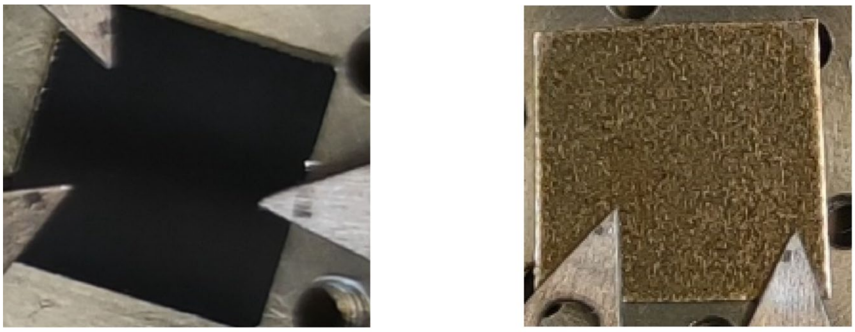

(a) ZrO_2 film/Si wafer (b) CoO film/unpolished Co foil

Figure S8 Photographs of (a) a ZrO_2 film on a flat Si wafer prepared by atomic layer deposition and (b) a CoO film on an unpolished, rough Co foil obtained by oxidation in $1x10^{-5}$ mbar O_2 at 600 K for 1 h.

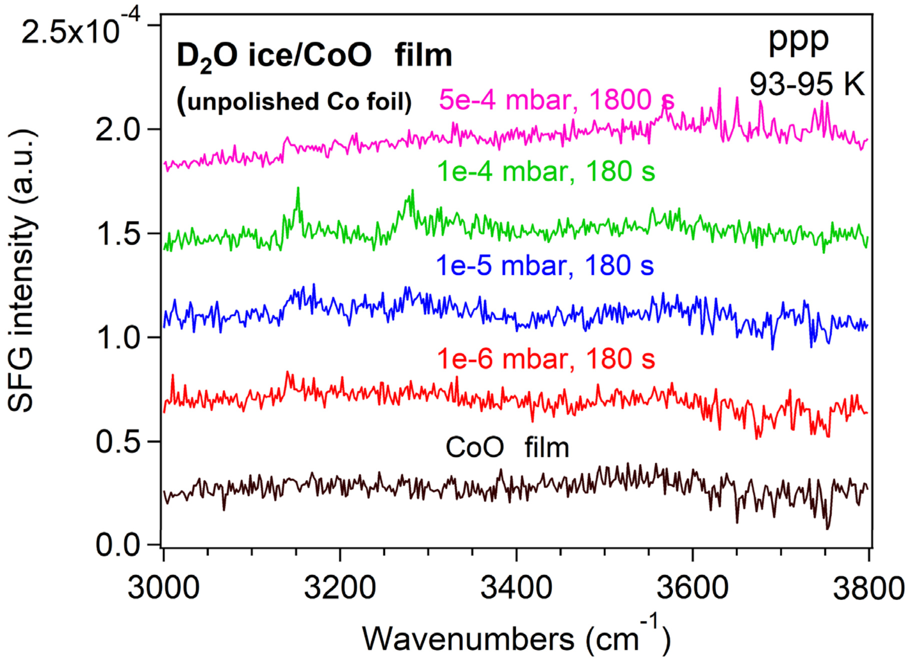

Figure S9 ppp-SFG spectra of D_2O adsorption on CoO/Co foil (unpolished) at 93-95 K in the range of 3000-3800 cm^{-1}.

References

[1] Y. Cao; L. Nyborg; U. Jelvestam. *Surf. Interface Anal.* 2009, **41**, 471–483. https://doi.org/10.1002/sia.3050.

[2] W. D., and S. Yip. *Materials interfaces: Atomic-level structure and properties*; Chapman and Hall, 1992.

[3] M. C. Biesinger; B. P. Payne; A. P. Grosvenor; L. W. M. Lau; A. R. Gerson; R. S. C. Smart. *Appl. Surf. Sci.* 2011, **257**, 2717–2730. https://doi.org/10.1016/j.apsusc.2010.10.051.
